# *Neisseria gonorrhoeae*: mechanisms of immune evasion, antimicrobial resistance, and vaccine development challenges

**DOI:** 10.3389/fmicb.2026.1824540

**Published:** 2026-06-16

**Authors:** Ilaria Onofrio, Calman A. MacLennan

**Affiliations:** 1 Nuffield Department of Medicine, The Jenner Institute, University of Oxford, Oxford, United Kingdom; 2 Department of Immunology and Immunotherapy, School of Medical and Dental Sciences, University of Birmingham, Birmingham, United Kingdom

**Keywords:** antimicrobial resistance (AMR), gonococcal vaccines, gonorrhoea, immune evasion, *Neisseria gonorrhoeae*

## Abstract

*Neisseria gonorrhoeae* is a strictly human-adapted Gram-negative bacterium and the causative agent of gonorrhoea, a sexually transmitted infection of major global health importance. Disease control is increasingly compromised by the rapid emergence of resistance to multiple antimicrobial classes and by the failure of natural infection to induce durable protective immunity. The ability of *N. gonorrhoeae* to persist within the host is driven by genetic and phenotypic adaptation strategies, including extensive antigenic and phase variation of surface-exposed components and a diverse array of immune evasion strategies. These include resistance to complement-mediated killing, manipulation of phagocytic cell function, suppression of adaptive immune responses, and mechanisms that overlap with antimicrobial resistance pathways. Experimental infection models have provided important insights into gonococcal pathogenesis and host immunity. Advances in vaccine research, including evidence of partial protection conferred by *N. meningitidis* group B outer membrane vesicle-based vaccines, have renewed interest in conserved gonococcal antigens and their use in multicomponent vaccine platforms. Identification of target antigens and immune pathways that confer protective immunity remain central to the development of effective vaccines against gonorrhoea and associated antimicrobial resistance.

## 1 *Neisseria gonorrhoeae* biology

Belonging to the *Neisseria* genus within the Betaproteobacteria, *Neisseria gonorrhoeae* is a Gram-negative diplococcus responsible for gonorrhoea, the second most frequently reported sexually transmitted infection (STI) worldwide (Hung and Christodoulides, 2013; Costa-Lourenço et al., 2017). While most of the 20-plus *Neisseria* species are commensal, non-harmful inhabitants of warm-blooded host mucous membranes, such as the human upper respiratory tract (Bovre and Holten, 1970; Knapp and Hook, 1988; Liu et al., 2015), *N. gonorrhoeae* and *N. meningitidis* are pathogenic to humans (Virji, 2009).

Like other Gram-negative bacteria, the cell wall of gonococci consists of a thin peptidoglycan layer located in the periplasmic space, with this latter enclosed between the inner cytoplasmic membrane and the outer membrane (Silhavy et al., 2010). Despite the high genetic similarity between *N. gonorrhoeae* and *N. meningitidis*, sharing 80–90% sequence homology (Tinsley and Nassif, 1996; Hadad et al., 2012), *N. gonorrhoeae* notably lacks the genes to synthesise a Group II polysaccharide capsule, a crucial virulence factor for *N. meningitidis* survival within the host (Virji, 2009; Rouphael and Stephens, 2012). These two pathogenic *Neisseriae* also typically colonise different niches, leading to distinct disease manifestations. *N. meningitidis* usually resides asymptomatically in the nasopharynx, potentially causing invasive diseases, like meningitis or septicaemia in cases of genetic predisposition, exposure to new strains, or immunocompromise (Pollard and Frasch, 2001; Virji, 2009), while *N. gonorrhoeae* primarily infects the urogenital tract, rectum, throat, and eyes, causing more localised inflammation and lower mortality (Unemo et al., 2019b). Nonetheless, both pathogens employ antigenic and/or phase variation mechanisms to alter their surface antigens, thereby enabling evasion from the host immune system (van der Woude and Bäumler, 2004).

### 1.1 *N. gonorrhoeae* genetic flexibility

*N. gonorrhoeae* has remarkable genetic flexibility which underpins its capacity for rapid adaptation, immune evasion, and persistence. A key mechanism is natural competence, enabling gonococci to acquire exogenous DNA from their surroundings throughout all growth phases. This mechanism is mediated by Type IV pili, hair-like appendages that facilitate the horizontal transfer of genetic traits between strains, including genes encoding antibiotic resistance or virulence factors, thereby complicating clonal analysis of multiple gonococcal strains (Hamilton and Dillard, 2006; Unemo and Dillon, 2011). Non-palindromic DNA uptake sequences (DUS), such as 5′-GCCGTCTGAA-3′ and 5′-ATGCCGTCTGAA-3′, act as recognition signals for selective DNA acquisition and homologous recombination (Spencer-Smith et al., 2016). Gonococci also use conjugation for plasmid (but not chromosomal DNA) transfer between bacterial cells via direct physical contact, also mediated by pili, further spreading genetic material including antibiotic resistance genes (Sox et al., 1978; Pachulec and van der Does, 2010).

Beyond competence-mediated transformation, *N. gonorrhoeae* demonstrates significant genetic flexibility via the hypervariability of its major surface antigens. Some gonococcal surface-exposed elements, such as proteins or lipooligosaccharide (LOS), can undergo phase and antigenic variation (Swanson et al., 1992). Phase variation is a reversible process characterised by high-frequency switching of gene expression (on or off) in individual bacterial cells, often caused by slipped-strand mispairing during DNA replication at repetitive sequences, leading to frameshifts or promoter disruptions (van der Woude and Bäumler, 2004). Antigenic variation involves low-frequency recombination, resulting in the expression of functionally conserved but alternative forms of an antigen (van der Woude and Bäumler, 2004; Deitsch et al., 2009). These dynamic genetic mechanisms are intrinsically linked to the structural components of the *N. gonorrhoeae* cell, particularly its surface-exposed elements.

### 1.2 Structural elements of *N. gonorrhoeae* cell wall

The *N. gonorrhoeae* outer surface is adorned with several key structural virulence factors:

#### 1.2.1 Pili

These hair-like appendages, primarily Type IV pili, facilitate initial adherence to epithelial cells and enable bacterial movement through twitching motility (Punsalang and Sawyer, 1973). Twitching motility is driven by the extension and retraction of the pili, which attach to a surface, and then retract, pulling the bacterial cell forward. Pili consist of a primary pilus subunit, known as pilin (PilE), and an adherence-associated protein (PilC) found at the tip of the pilus as well as inside the bacterium (Jonsson et al., 1991). Pili connect the bacterium to the external environment, enabling attachment to human epithelial cells *in vitro* (Merz and So, 2000) and facilitating biofilm formation and DNA uptake (Shaughnessy et al., 2019).

Gonococci can switch frequently between piliated and non-piliated forms (Punsalang and Sawyer, 1973), and the majority of gonococci recovered from symptomatic natural infections are piliated (James and Swanson, 1978). Pili phase variation is mainly driven by changes in the pilin structural gene, which result in the production of defective pilin proteins (Bergström et al., 1986). The PilE subunits of *N. gonorrhoeae* can undergo antigenic variation by recombination between the *pilE* gene and a silent *pilS* gene (Haas et al., 1992). This mechanism allows identical gonococcal strains to express different amino acid sequences and, therefore, different pilus types (Gotschlich, 1984).

#### 1.2.2 Lipooligosaccharide (LOS)

**Figure 1:**
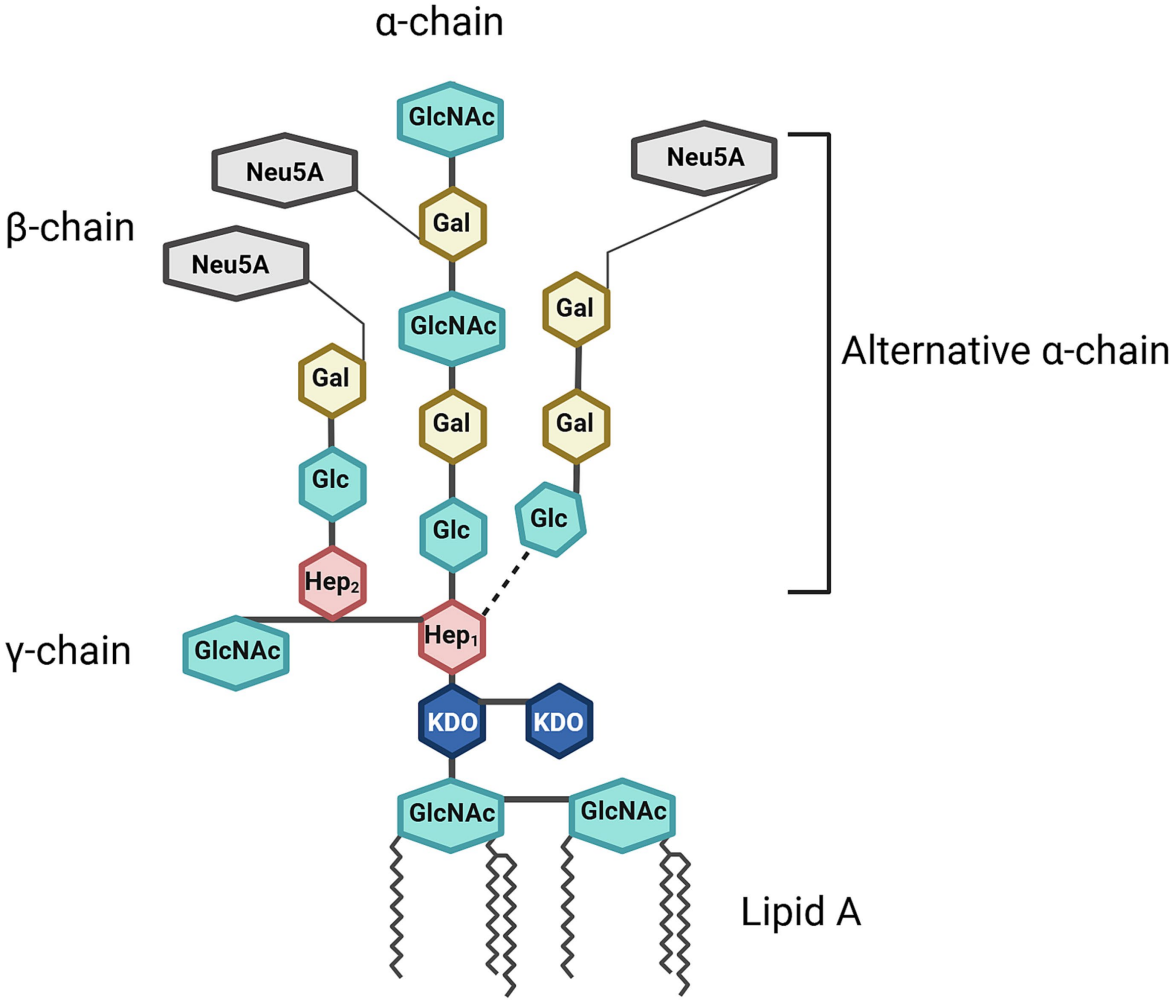
*N. gonorrhoeae* lipooligosaccharide (LOS) general structure. Gonococcal LOS consists of three oligosaccharide chains branching from two heptose (Hep) residues. These heptose residues are linked to lipid A through a 2-keto-3-deoxy-manno-octulosonic acid (KDO) molecule. One oligosaccharide chain extends from the first heptose (Hep 1), while two chains branch from the second heptose (Hep 2). The figure shows 2 alternative *α*-chains commonly expressed by gonococci. The LOS branching can be extended by the addition of hexoses or can be terminated by sialic acid (Neu5A) addition on galactose (Gal) residues. The lipid A component is constituted by two glucosamine molecules (GlcNAc), each with 3 acyl chains.

Gonococcal LOS is a large glycolipid comprising a hydrophobic lipid A domain and three oligosaccharide chains linked via 2-keto-3-deoxy-manno-octulosonic acid (KDO) sugars (Shaughnessy et al., 2019) (Figure 1). The lipid A structure is conserved within the pathogenic *Neisseria* species and consists of two glucosamine molecules, each with three acyl chains, bound to one of two KDOs (Figure 1). Unlike the lipopolysaccharide (LPS) of other Gram-negative bacteria, Neisserial LOS lacks a repeating polysaccharide (O-antigen) chain (Apicella et al., 1987).

Glycosyl transferase genes (*lgt*) regulate the synthesis of glycan chains, and some LOS assembly genes are phase variable, altering LOS size and structure (Yang and Gotschlich, 1996; Banerjee et al., 1998). Gonococci can also terminate (“cap”) the LOS with sialic acid (5-acetamido-2,4-dihydroxy-6-(1,2,3-trihydroxypropyl)oxane-2-carboxylic acid, referred to as Neu5A) by producing a sialyltransferase that utilises a host substrate, cytidine monophosphate-N-acetylneuraminic acid (CMP-NANA) (Mandrell et al., 1990) (Figure 1), mimicking host cells to evade immunity.

#### 1.2.3 Colony opacity-associated (Opa) proteins

Opa proteins are integral adhesin proteins located in the outer membrane, and their expression determines the appearance (opacity) of the colonies on agar plates. These proteins mediate attachment to epithelial cells and subsequent internalisation (Hauck and Meyer, 2003). Their expression is highly variable, driven by phase and antigenic variation, mediated by intragenic recombination (Hauck and Meyer, 2003). Gonococci typically encode 11 Opa proteins (Bhat et al., 1991), and an individual gonococcal cell can express none or several Opa proteins simultaneously (Swanson, 1978). These proteins can bind to two specific host factors. The majority bind to the carcinoembryonic antigen-related cell adhesion molecule (CEACAM), while a minority bind to the cell surface molecule heparan sulphate proteoglycan (HSPG) (Virji, 2000). Receptors for CEACAM are present on various cell types, including those lining the urogenital tract. The binding of Opa to CEACAM facilitates the attachment and entry of the bacteria into host cells, allowing them to invade and colonise mucosal surfaces (Liu et al., 2011).

#### 1.2.4 Porin ion channel protein (PorB)

Porin (PorB or Protein I) accounts for over 60% of the total gonococcal outer membrane protein by weight (Johnston et al., 1976) and is noncovalently associated with reduction-modifiable protein (Rmp) (Johnson et al., 1977). PorB is essential for gonococcal viability, facilitating the transport of small nutrients into the bacterium and the removal of waste products (Chen and Seifert, 2013). There are two major subtypes, encoded by the mutually exclusive alleles, PorB_IA_ and PorB_IB_,(Morello and Bohnhoff, 1989; van Putten et al., 1998). Therefore, a gonococcal isolate can express only one of the two PorB protein isotypes, and their expression is used as a basis for gonococcal typing. Gonococcal strains of the PorB_IA_ isotype are often associated with more invasive disease states and tend to resist complement-mediated bacteriolysis. Strains expressing PorB_IB_ are mostly isolated from patients with localised gonococcal infections (van Putten et al., 1998).

## 2 Gonorrhoea pathogenesis and immunology

### 2.1 Clinical manifestation

The bacterium predominantly infects the urogenital tract epithelia, with the cervix in women and the urethra in men being common sites of infection (Detels et al., 2011; Mitchell and Prabhu, 2013). However, it can infect other mucosal sites, including the oropharynx, anorectum, and occasionally the eyes (Noble et al., 1979; Lee et al., 2002; Danby et al., 2016; Gottlieb et al., 2020). Data from the World Health Organization (WHO) in 2020 estimated approximately 82.4 million [95% UI: 47.7–130.4 million] new cases of gonorrhoea globally among individuals aged 15–49 years (WHO, 2021), with over 90% of cases occurring in low- and middle-income countries (LMICs), which are disproportionally affected (Rowley et al., 2019).

Approximately 90% of men infected with gonococcus develop noticeable symptoms 2 to 7 days after exposure, including dysuria and urethral discharge, characterised by a neutrophil influx, which is insufficient to clear the infection. In contrast, 30–50% of infected women are asymptomatic or have symptoms that go unrecognised (Evans, 1977; Edwards et al., 2000; Quillin and Seifert, 2018). Rectal infections are often asymptomatic and most frequently occur among men who have sex with men (MSM) (Miller, 2006). Symptoms in women can include vaginal and cervical discharge, lower abdominal or pelvic pain, dyspareunia, and dysuria. Prolonged untreated gonococcal infections can cause serious and permanent reproductive health outcomes in women. Vaginal gonococcal infections can ascend to the uterus or fallopian tubes and cause pelvic inflammatory disease (PID). PID can result in internal abscesses, persistent pelvic pain, and damage to the fallopian tubes, which may lead to infertility. Additionally, gonococcal infection increases the risk of ectopic pregnancy (Unemo et al., 2019b; Xu and Gray-Owen, 2021).

Disseminated gonococcal infections (DGI) are uncommon and develop when gonococci migrate into the bloodstream and spread to distant sites in the body. Systemic infection can occur due to uncontrolled bacterial growth in the bloodstream and lead to the development of localised septic arthritis and arthritis-dermatitis syndrome (Eisenstein and Masi, 1981; Masi and Eisenstein, 1981; Fidalgo et al., 2023; Humayun et al., 2023). Late clinical manifestations of extra-pelvic gonorrhoea can include endocarditis and pericarditis (Thompson and Brantley, 1996; Said and Tirthani, 2021). Pregnant women who are infected can pass the infection to their newborns during childbirth, affecting the conjunctival mucosa and causing ophthalmia neonatorum, which can lead to permanent eye damage (Smith and Halse, 1955). Gonococcal infection is also linked to an increase in HIV acquisition and transmission (Fleming and Wasserheit, 1999; Johnson and Lewis, 2008).

While *N. gonorrhoeae* generally causes low-mortality disease, its significant public health burden is intensified by high morbidity, especially in women and untreated cases, along with bacterial antigenic variability and rapid development of antimicrobial resistance (AMR), complicating both treatment and prevention efforts.

### 2.2 Establishment of infection

The initial step in gonococcal infection involves the bacteria adhering to epithelial cells, a process primarily mediated by several surface components including type IV pili, LOS, Opa proteins, and PorB (Winther-Larsen et al., 2001; Edwards and Butler, 2011; Quillin and Seifert, 2018). Among these, type IV pili play a crucial role in attaching to host cells, although the exact host receptors facilitating this interaction are uncertain. The host proteins CD46, CR3, and I-domain-containing integrins have been identified as receptors for pili. The binding of gonococcal pili to CR3 requires the bacterium to be opsonised with iC3b, a complement protein formed by the cleavage of C3b by factor I, and also depends on the presence of porins (Jennings et al., 2011). CR3 is expressed on cervical epithelial cells but absent on male urethral epithelial cells (Källström et al., 1997), suggesting that interactions with gonococcal pili in the male urethra are more likely mediated by CD46 and I-domain-containing integrins (Walker et al., 2023). CD46 has been confirmed as a receptor for pilus-mediated attachment in various cell types *in vitro* (Källström et al., 1997; Gill and Atkinson, 2004; Kirchner et al., 2005), although CD46-independent mechanisms, possibly involving I-domain-containing integrins, may also contribute to pilus binding (Tobiason and Seifert, 2001; Edwards and Apicella, 2005; Kirchner et al., 2005).

**Figure 2:**
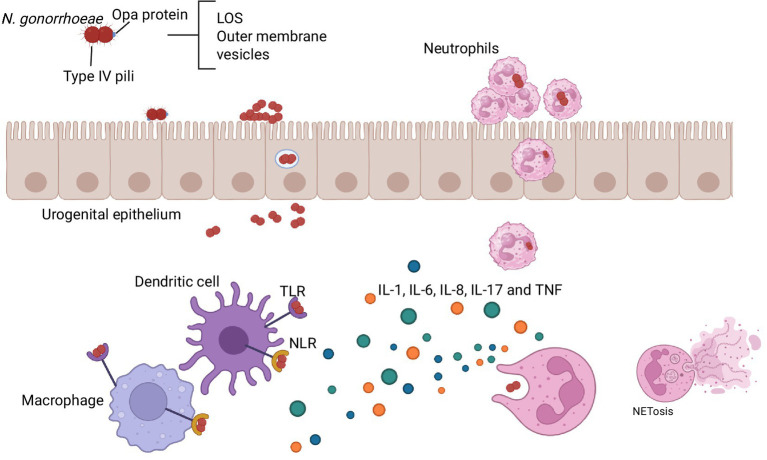
*N. gonorrhoeae* infection. The initial infection with gonococcus is mediated by the attachment of gonococci to host epithelial cells via type IV pili and Opa proteins. Following this initial adherence, the bacterium replicates and can undergo transcytosis. During these early stages, *N. gonorrhoeae* sheds lipooligosaccharide (LOS) and outer membrane vesicles (OMVs), which activate innate immune cells. This activation triggers the release of pro-inflammatory cytokines, leading to the recruitment of large numbers of neutrophils from the bloodstream to the infection site. Neutrophils phagocytose gonococci and release neutrophil extracellular traps (NETs). TLR Toll-like receptor, NLR NOD-like receptor.

Following pili-mediated attachment, pili retract, drawing the bacterium closer to the epithelial surface. Subsequent interactions, mediated by Opa-CEACAM and Opa-HSPG binding, stabilize attachment and initiate invasion. *N. gonorrhoeae* can then replicate on the epithelial surface and penetrate underlying tissues through transcytosis (Quillin and Seifert, 2018). In the early stages of infection, gonococci release LOS fragments and outer membrane vesicles (OMVs), which engage with the Toll-like receptors (TLR) and nucleotide-binding oligomerisation domain (NOD)-like receptors in epithelial cells, phagocytic cells and antigen-presenting cells (APCs) (Singleton et al., 2005; Escobar et al., 2018). This engagement initiates a cascade of intracellular signalling pathways (e.g., nuclear factor kappa B (NF-κB) activation) that culminate in the robust production and secretion of pro-inflammatory cytokines and consequently a large neutrophil response (Figure 2). Neutrophils are drawn to the infection site and aim to eradicate the bacteria through processes such as phagocytosis, production of reactive oxygen species, secretion of cationic peptides and various antimicrobial enzymes (Quillin and Seifert, 2018). However, *N. gonorrhoeae* has evolved mechanisms to survive and replicate within neutrophils and epithelial cells, evade antimicrobial functions, modulate apoptosis, and resist antibody-dependent complement-mediated killing, allowing it to survive and persist despite host immune responses (Chen and Seifert, 2011; Criss and Seifert, 2012; Château and Seifert, 2016).

### 2.3 Adaptive immunity to *N. gonorrhoeae*

Re-infections with *N. gonorrhoeae* are common, indicating that a primary gonorrhoea infection generally fails to elicit a protective immune response (Russell et al., 2020). This failure is possibly the result of weak or dysregulated immune responses at the mucosal surface, which do not develop into durable or effective systemic immunity. Natural gonococcal infections have been reported to induce a modest increase in both blood and mucosal gonococcal-specific antibodies (Hedges et al., 1998; Hedges et al., 1999; Russell et al., 2020). However, immune responses to gonococcus are often heterogeneous between individuals and complicated by pre-existing antibodies to commensal *Neisseria* spp. surface antigens in pre-infection sera, which can confound results (Lovett and Duncan, 2018; Gulati et al., 2019c).

Early studies demonstrated that serum bactericidal antibodies against gonococcus may develop in subjects with more severe manifestations of the disease, such as DGI and salpingitis (inflammation of the fallopian tubes) (Kasper et al., 1977; Hook et al., 1984). In a clinical study of men with uncomplicated gonococcal urethritis, serum bactericidal antibodies against the infecting strain were detected in only 31% of individuals prior to treatment and were absent in uninfected women when tested against PID-associated gonococcal strains. Among women with PID, bactericidal antibodies were generally absent during acute infection but developed during convalescence in a subset of patients with severe disease (70%). Only 12.5% of the women who had mild or moderate illness exhibited a similar increase in antibody levels, suggesting a correlation between the severity of the disease and the development of bactericidal antibodies (Kasper et al., 1977).

Bactericidal antibodies function primarily through complement activation, which initiates the formation of the membrane attack complex (MAC). The importance of complement in immunity against gonococcus is indicated by individuals with immunodeficiencies affecting terminal complement components, which impair the formation of the C5b-9 MAC, having high susceptibility to DGI (Ram et al., 2010; Lewis and Ram, 2020).

Several human studies have characterised antigen-specific antibody responses following gonococcal infection. Early immunoblotting and ELISA analyses detected antibodies against LOS, PorB, and pili in subsets of infected individuals. However, these responses were inconsistent and did not correlate with protection from reinfection. In a small cohort study (13 subjects), antibodies against PorB (12/13) and LOS (9/13) or both (8/13) were detected in the majority of infected individuals (Hicks et al., 1987). Consistent with these findings, elevated anti-pilus antibody levels were reported in men with gonococcal urethritis and in women with cervicitis compared with uninfected controls, with the highest titres observed in women with gonococcal pelvic inflammatory disease compared with those with non-gonococcal PID (Miettinen et al., 1989).

High-resolution antibody profiling approaches have recently been applied to human cohorts with repeated gonococcal exposure. A microarray-based study of female sex workers in Kenya characterised IgG and IgA responses against a broad panel of gonococcal antigens and demonstrated highly heterogeneous antibody repertoires following natural infection, with limited overlap between individuals and poor persistence over time (Stejskal et al., 2024). Overall, these studies indicate that natural gonococcal infection can elicit antigen-specific antibody responses, albeit inconsistently and without clear evidence of protection.

Although reinfections are common, some evidence of specific but incomplete serovar immunity (defined as immunity to strains sharing the same PorB type) was reported among sex workers in a longitudinal study in Nairobi, Kenya. In this study, women who contracted a particular gonococcal serovar., identified by the presence of antibodies against a specific PorB type, experienced a 2- to 10-fold reduction in the risk of subsequent infection by the same serovar, except for one specific PorB type, 1B-1 (Plummer et al., 1989). In this study, the duration of prostitution was negatively correlated with the incidence of infection, suggesting that protective immunity to gonococcus develops over time in these individuals, possibly due to repeated exposure to a range of gonococcal strains expressing different Por-types (Plummer et al., 1989). These findings are consistent with earlier research indicating that the likelihood of recurrent gonococcal salpingitis is reduced when women were exposed to gonococcal strains of the same serovar (Buchanan et al., 1980). Both studies detected antibodies against PorB and Opa. While specific immunity to individual serovars has been demonstrated, the diversity of PorB types and other antigenic variants of *N. gonorrhoeae* makes it difficult to attain complete and lasting protection against different strains.

In addition to antibody responses, gonococcal infection elicits antigen-specific T cell responses that are specific to gonococcal antigens (Lovett and Duncan, 2018). Gonococcal infections have been associated with significant alterations in cytokine profiles, including elevated levels of serum IL-17, IFN-*γ*, and IL-23 in affected patients compared to healthy individuals (Gagliardi et al., 2011). However, the immune response is dominated by a Th17 response, characterised by elevated IL-17 levels, while simultaneously downregulating Th1 responses, as evidenced by relatively lower levels of IFN-γ (Gagliardi et al., 2011). Elevated levels of IL-17 and other inflammatory cytokines are also detected in the cervical secretions of infected women, reinforcing the association of IL-17 with gonococcal infection (Masson et al., 2015).

In summary, the lack of protective immunity to *N. gonorrhoeae* following infection is poorly understood and presents a challenge to vaccine development. This is likely influenced by bacterial genetic variability, resulting in antigenic variation which limits the breadth of immunity induced by an episode of gonorrhoea; immune evasion strategies employed by the bacterium; host immune responses that are short-lived, poorly protective, and insufficient to generate durable immunological memory. While natural gonococcal infections can induce modest increases in gonococcal-specific antibodies, it appears that antibody responses are typically low until the infection extends to the upper genital tract, suggesting that local infections alone may not elicit a robust immune response sufficient for protection (Hedges et al., 1999).

### 2.4 *N. gonorrhoeae* immune evasion mechanisms

*Neisseria gonorrhoeae* has several mechanisms to evade the host immune system. These mechanisms include the production of IgA protease, induction of blocking antibodies and different ways of resisting complement-mediated killing and killing by phagocytosis (Belcher et al., 2023).

#### 2.4.1 IgA protease production

All pathogenic *Neisseria,* but not commensal *Neisseria*, express and secrete the proteolytic enzyme, IgA1 protease, which can cleave human serum and secretory IgA1 (Plaut et al., 1975; Pohlner et al., 1987). Elimination of IgA1, helps the bacterium evade immune defence at mucosal surfaces, where it can then establish an initial infection.

#### 2.4.2 Induction of blocking antibodies by reduction modifiable protein (Rmp)

Both pathogenic *Neisseria* species express Rmp, a highly conserved outer membrane protein (Judd, 1982). Rmp is not phase or antigenically variable and can induce anti-Rmp antibodies following natural gonococcal infection. These anti-Rmp antibodies inhibit the action of bactericidal antibodies targeting LOS and PorB by restricting complement availability, potentially increasing susceptibility to reinfection (Joiner et al., 1985; Plummer et al., 1993; Rice et al., 1994; Gulati et al., 2015a).

#### 2.4.3 Recruitment of complement inhibitors

*N. gonorrhoeae* has developed strategies to evade complement, including the expression of host-like molecules and attracting complement inhibitors to its surface.

**Figure 3:**
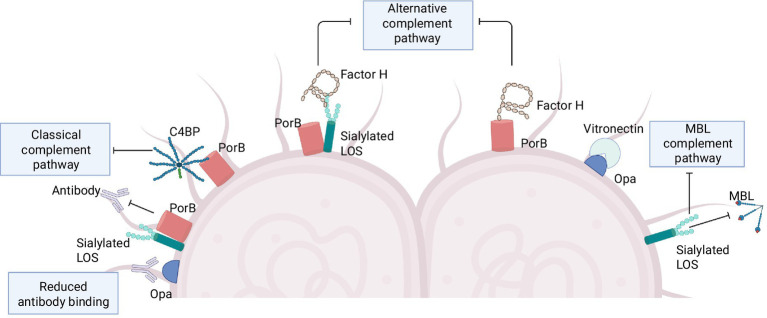
Complement-mediated killing evasion systems employed by *N. gonorrhoeae*. Gonococci avoid complement-mediated killing through multiple strategies, including the recruitment of host complement inhibitors such as Factor H (fH) and C4b-binding protein (C4BP), as well as by mimicking host cells through sialylation of its LOS. MBL mannose binding lectin. Figure adapted from Quillin and Seifert, 2018.

The gonococcus scavenges the host substrate CMP-NANA to sialylate its LOS. Sialic acid is commonly found on the surface of host cells, where it plays a role in regulating immune responses and protecting host tissues from complement-mediated damage (Ngampasutadol et al., 2008; Kajander et al., 2011). By incorporating sialic acid onto its LOS, gonococcus mimics the sialylation pattern of host cells and evades complement recognition. Sialylation of the LOS and the presence of PorB also allow the bacterium to recruit complement inhibitor factor H (fH) (Madico et al., 2007). The recruitment of fH prevents C3b deposition via the alternative complement pathway, thereby blocking the formation of the C3 convertase and enhancing resistance to complement-mediated killing (Parsons et al., 1992; Smith et al., 1992; Lewis and Ram, 2020). The binding of fH by gonococci is specific to human fH, and helps account for the restriction of gonococcal infection to humans (Lewis and Ram, 2020). Sialylation of LOS also reduces IgG binding to PorB and inhibits Mannose Binding Lectin (MBL) attachment, thereby inhibiting the activation of the classical and MBL complement pathways (Devyatyarova-Johnson et al., 2000; Gulati et al., 2015b) (Figure 3).

Gonococci recovered from male urethral secretions exhibit complete resistance to serum-mediated killing. However, some strains lose their ability to resist complement-mediated killing after a single subculture *ex vivo*. This loss of resistance is directly linked to the absence of sialic acid, and when sialic acid is added back to the growth media, these strains regain their serum resistance (Ward et al., 1970).

PorB directly interacts with fH and C4 binding protein (C4BP), which inhibits the classical and lectin pathways (Ram et al., 1998) (Figure 3), while Opa protein can bind vitronectin, inhibiting complement-mediated bactericidal activity by preventing C9 polymerisation into the C5b-9 MAC (Figure 3) (Duensing and Putten, 1998).

#### 2.4.4 Manipulation of phagocytosis by neutrophils and macrophages

**Figure 4:**
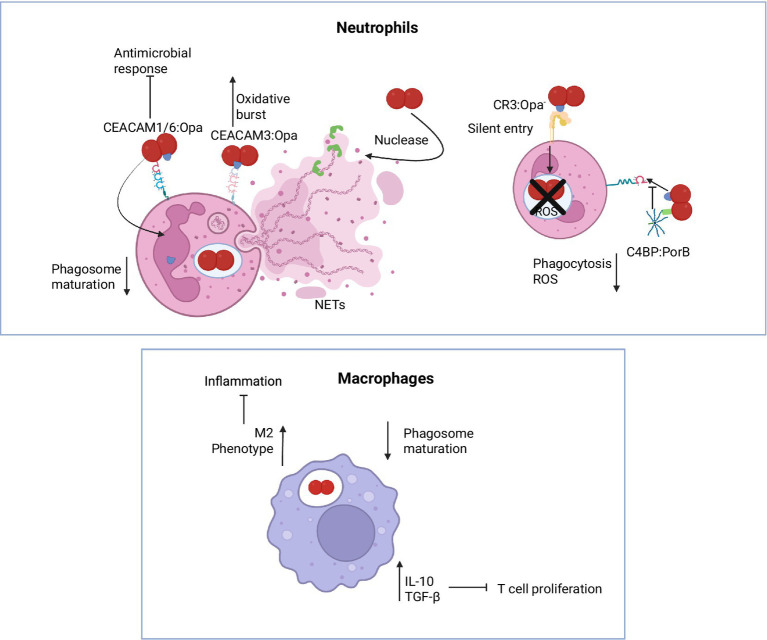
*N. gonorrhoeae* manipulation of phagocytosis by neutrophils and macrophages. Neutrophils, upon encountering gonococci, attempt to neutralise the bacteria through neutrophil extracellular nets (NETs), reactive oxygen species (ROS), and phagocytosis. Opa-expressing gonococci bind to CEACAM3 receptors on neutrophils, which leads to the internalisation and killing of the bacteria. Opa-CEACAM1 or CEACAM6 interaction induces internalisation without the generation of an antimicrobial response. Opa-positive gonococci utilise binding to C4BP to suppress ROS production and prevent phagocytosis. Opa-negative gonococci are internalised through CR3 binding, leading to ‘silent entry’. Once internalised, the bacteria manipulate phagosome maturation by delaying fusion with primary granules. *N. gonorrhoeae* promotes non-inflammatory uptake by macrophages and manipulation of macrophage differentiation towards the M2 phenotype. Following internalisation, the bacteria interfere with phagolysosomal fusion and therefore phagosome maturation. Gonococci also modulate macrophage cytokine responses by increasing IL-10 and TGF-β production, inhibiting T cell proliferation.

Gonococcal infection involves significant neutrophil recruitment, yet this innate immune response often fails to clear the infection (Belcher et al., 2023). Neutrophils kill gonococci through phagocytosis and by releasing neutrophil extracellular traps (NETs). Once phagocytised by neutrophils, bacteria are enclosed in a phagosome that contains reactive oxygen species (ROS), degradative enzymes, and antimicrobial peptides, all of which interact to kill the pathogen. Opa-expressing gonococci bind to CEACAM expressed by human neutrophils, which mediate the internalisation of the bacteria within phagocytes (Boulton and Gray-Owen, 2002). Neutrophils express three CEACAM receptors: CEACAM1, CEACAM3, and CEACAM6. While Opa binding to CEACAM3 triggers internalisation and an oxidative burst leading to bacterial killing, Opa interaction with CEACAM1 or CEACAM6 leads to internalisation without stimulating ROS production or toxic granule release, aiding bacterial survival (Sarantis and Gray-Owen, 2012) (Figure 4). Additionally, Opa-positive gonococci can exploit their binding to C4BP, described above, in a complement-independent mechanism to suppress the production of ROS by neutrophils and prevent phagocytosis (Werner et al., 2023).

In addition to the CEACAM route, neutrophils can internalise Opa-negative gonococci through CR3 binding. This CR3-mediated internalisation is associated with a “silent entry,” where the neutrophils remain in a less activated state, reducing their ability to kill gonococci (Smirnov et al., 2023) (Figure 4).

Once internalised, gonococci influence phagosome maturation by delaying fusion with primary granules, enhancing intracellular survival. *N. gonorrhoeae* can also alter neutrophil apoptosis, delaying or modifying the process to aid persistence (Johnson and Criss, 2013) (Figure 4). The ability of *N. gonorrhoeae* to manipulate phagosome maturation does not require the bacterium to be actively metabolising. Heat-inactivated *N. gonorrhoeae*, when exposed to neutrophils, still influence the maturation of the phagosome, suggesting bacterial surface components are involved (Johnson and Criss, 2013; Smirnov et al., 2014). Gonococci can also alter neutrophil apoptosis by delaying or modifying the normal apoptotic process, aiding persistence in the host (Simons et al., 2006; Chen and Seifert, 2011), and even “hitchhike” on migrating neutrophils via pili, by attaching to the uropod, utilising their movement to traverse epithelial barriers (Söderholm et al., 2011).

Macrophages, crucial for immunity and tissue homeostasis, can also be manipulated by the bacterium (Figure 4). Once phagocytosed by macrophages, *N. gonorrhoeae* can survive and replicate within these cells (Château and Seifert, 2016) and manipulate macrophage differentiation into anti-inflammatory M2 macrophages, by promoting anti-inflammatory cytokines like IL-10 and TGF-*β*. IL-10 suppresses pro-inflammatory cytokines and shifts macrophage polarisation, reducing M1-associated surface markers and co-stimulatory molecules, impairing antigen presentation and T cell activation, and thereby promoting gonococcal survival (Mills, 2012; Ortiz et al., 2015).

#### 2.4.5 Efflux pump expression as a mechanism of antimicrobial resistance and immune evasion

*N. gonorrhoeae* express up to five efflux pump systems, which actively transport a wide range of substances, including antimicrobials. The major efflux pumps are the MtrCDE, FarAB-MtrE, NorM, MtrF and MacAB-MtrE systems (Costa-Lourenço et al., 2017; Handing et al., 2018; Unemo et al., 2019b).

Among these, the MtrCDE efflux pump is primarily responsible for expelling a wide range of hydrophobic agents, as well as several antibiotics such as penicillin and tetracycline (Hagman et al., 1995; Warner et al., 2008), and host-derived antimicrobial peptides present in NETs, protecting gonococci from both extracellular killing in NETs and exposure to granule contents (Warner et al., 2008; Lappann et al., 2013; Handing et al., 2018). Similarly, the MtrF efflux pump expels hydrophobic agents and certain antibiotics, particularly sulphonamides (Veal and Shafer, 2003). The FarAB-MtrE efflux pump is involved in removing long-chain fatty acids and antimicrobial fatty acids, contributing to resistance to these compounds (Lee and Shafer, 1999). The MacAB-MtrE efflux system is associated with resistance to macrolide antibiotics, such as erythromycin and azithromycin (Rouquette-Loughlin et al., 2005). The NorM efflux pump exports a variety of compounds, including fluoroquinolone antibiotics (Rouquette-Loughlin et al., 2003). Overall, the efflux pump systems are central to the ability of gonococcus to resist multiple antibiotics and host defences, including neutrophil-mediated killing via NETs, degranulation products, and mucosal antimicrobial peptides/lipids, thereby posing significant challenges to effective treatment.

#### 2.4.6 Iron- and zinc-regulated proteins allow bacterial survival in hostile environments

Iron and zinc are essential nutrients for gonococci and play critical roles in bacterial metabolism and virulence. *N. gonorrhoeae* expresses several key proteins to acquire iron from the host, especially under iron-limited conditions including Transferrin-binding proteins A and B (TbpA and TbpB), Lactoferrin-binding proteins A and B (LbpA and LbpB), Haemoglobin-haptoglobin utilisation protein A and B (HpuA and HpuB), and Ferric enterobactin transporter FetA.

TbpA extracts iron from transferrin with the help of TbpB, which increases the efficiency of the transferrin binding (Cornelissen et al., 1992; Anderson et al., 1994). Similar functions are covered by LbpA and B, which utilise lactoferrin substrate (Mickelsen et al., 1982). HpuA and B are components of the haem-iron acquisition system, binding haemoglobin and its complexes (Lee, 1992). Gonococci, like other gram-negative bacteria, can also use the siderophore enterobactin, secreted by members of the family Enterobacteriaceae, as a source of iron through the binding of FetA to the ferric enterobactin complexes (Carson et al., 1999). Once iron is acquired, ferric binding protein A (FbpA) shuttles it from the membrane to the cytoplasm of the bacterium, ensuring a continuous supply of iron for metabolic processes (Parker Siburt et al., 2012).

Zinc acquisition is crucial for enzyme activity, protein structure stabilisation, regulation of gene expression, and other bacterial activities. Gonococci acquire zinc via its transport system ZnuABC (Chen and Morse, 2001). Humans restrict zinc to infection sites by using zinc-sequestering proteins, including calprotectin (Stríz and Trebichavský, 2004). Calprotectin is released by neutrophils in the NETs, where it acts as an antimicrobial through its ability to bind zinc (Johnstone and Herzberg, 2022). However, *N. gonorrhoeae* can use the zinc-binding proteins TdfJ and TdfH to survive in zinc-depleted environments (Ray et al., 2022). These proteins bind, respectively, to calprotectin (Jean et al., 2016), and S100A7, another host protein that sequesters zinc (Maurakis et al., 2019), enabling gonococci to extract zinc from the host, thereby enhancing survival in NETs (Jean et al., 2016; Maurakis et al., 2019).

## 3 *N. gonorrhoeae* infection models

As *N. gonorrhoeae* is a pathogen that exclusively infects humans, studying its biology and pathogenesis using animal models presents considerable challenges. Currently, there are two experimental models available to characterise gonococcal genital tract infections: a controlled human infection model (CHIM) in men and a *β*-oestradiol-treated female mouse model.

### 3.1 Controlled human infection model (CHIM)

In the 1980s, an *N. gonorrhoeae* male urethritis CHIM was developed by investigators at the Walter Reed Army Institute of Research and University of North Carolina. This model involves inoculating gonococci in the urethra of male volunteers (Hobbs et al., 2011). Limited to male subjects, due to the risk of ascending gonococcal infections in women, the model enables a comprehensive evaluation of disease symptoms, as well as microbiological and immunological responses. The CHIM is potentially valuable for testing new vaccine candidates on a limited number of participants compared to larger numbers required for field efficacy trials. Furthermore, it provides a controlled environment to study immune responses, allowing the consideration of variables such as host genetics, phenotype, and medical history, and enables comparisons between infected and uninfected individuals (Waltmann et al., 2022). However, the model has limitations. First, the model relies on infection with a single gonococcal strain, typically a laboratory-adapted strain, which does not represent the extensive antigenic and genetic diversity of circulating *N. gonorrhoeae* strains. Second, the exclusive use of male subjects restricts the context of findings to males, and not females who experience different clinical manifestations, such as PID, and may also have different immune responses to gonococcal infection. Furthermore, the duration of infection is constrained by either a set time period or the time to development of symptoms. These restrictions limit the length of the study and prevent the examination of more severe and chronic forms of gonorrhoea (Belcher et al., 2023).

Nevertheless, the model provides valuable insights into the role and expression dynamics of surface-exposed molecules, including iron acquisition receptors and pili. For instance, a CHIM study involving a mutant gonococcal strain lacking both transferrin and lactoferrin binding receptors found such a strain is unable to cause urethritis, suggesting iron acquisition by gonococci is essential for disease (Cornelissen et al., 1998). Another study investigating the role of pili in human infection revealed that gonococci are still able to colonise and cause disease even when pili are absent, suggesting that although pili may contribute to pathogenesis, they are not essential for colonisation of the male urethra (Hobbs et al., 2011).

While the *N. gonorrhoeae* CHIM can be valuable in early-stage vaccine testing by offering controlled environments for assessing immune responses and potential vaccine efficacy, its ability to predict vaccine efficacy in field studies is not clear. A pilin-based subunit vaccine developed in the 1980s showed promising results in CHIM studies, including strong antibody responses and protection against the infecting homologous strain. However, the vaccine failed to protect against disease in a field efficacy study involving high-risk US military personnel (Boslego et al., 1991). This failure was attributed to the extensive antigenic variation of the pilin protein, which was not fully understood at the time.

In summary, the gonococcal CHIM offers significant potential for preliminary vaccine and therapeutic research by enabling detailed studies in a controlled setting. However, its limitations, including restricted number of participants, use of a single infecting strain and short duration of infection, emphasise the importance of viewing the model as part of a broad clinical research and development approach, including field efficacy trials.

### 3.2 The oestradiol-treated mouse model of *N. gonorrhoeae* genital tract infection

Since gonococcus is a pathogen that causes disease exclusively in humans, developing animal models that closely replicate human infection has been challenging. In 1999, a mouse model was established by treating female mice with *β*-oestradiol, which prolongs the oestrus phase of the reproductive cycle (Jerse, 1999). To prevent overgrowth of commensal flora secondary to β-oestradiol treatment, antibiotics are also administered (Jerse, 1999; Jerse et al., 2011).

During the follicular phase in women and the oestrus phase in female mice, increased oestrogen creates an environment beneficial for reproduction. However, in these phases, sex hormones also suppress innate, humoral, and cell-mediated immunity, making the female more susceptible to acquiring STIs (Wira and Fahey, 2008; Wira et al., 2010). These hormonal changes can be exploited to use female mice as surrogate hosts for *N. gonorrhoeae*. This approach enabled the successful establishment of gonococcal infection in the lower genital tract, and more recently, upper genital tract of the mouse.

*N. gonorrhoeae* infection of BALB/c and C57BL/6 mice typically persists for an average of 10 to 12 days (Jerse et al., 2011), with some infections lasting up to 40 days before gonococci are naturally cleared (Rice et al., 2017). In pre-treated mice, as in humans, gonococci are able to colonise and replicate within the vaginal and cervical epithelial tissues, inducing a localised response characterised by macrophages and neutrophil influx, the latter only in BALB/c mice (Jerse, 1999). Similar to natural infection in humans, a specific gonococcal antibody response is induced in mice, in both the genital mucosa and blood, but this response does not provide protection against reinfection with the same strain (Song et al., 2008). Despite its utility for studying gonococcal infections, the model has several limitations when applied to vaccine development. Oestradiol and antimicrobial treatment likely impact the host immune response during infection. The use of hormones and antimicrobials to allow gonococcal colonisation of the mouse vaginal tract introduces additional differences from natural infection in humans and likely influences host immune responses, limiting the direct applicability of findings to gonorrhoea in humans (Waltmann et al., 2022). Infection is transient and localised to the female genital tract, which restricts the ability to study disseminated gonococcal infections. Moreover, many gonococcal surface ligands that facilitate adherence to and invasion of host cells bind to specific receptors which are exclusively found in humans (Jerse et al., 2011), such as human CD46 receptor, CEACAMs and CR3, as well as host-specific factors such as transferrin.

**Figure 5:**
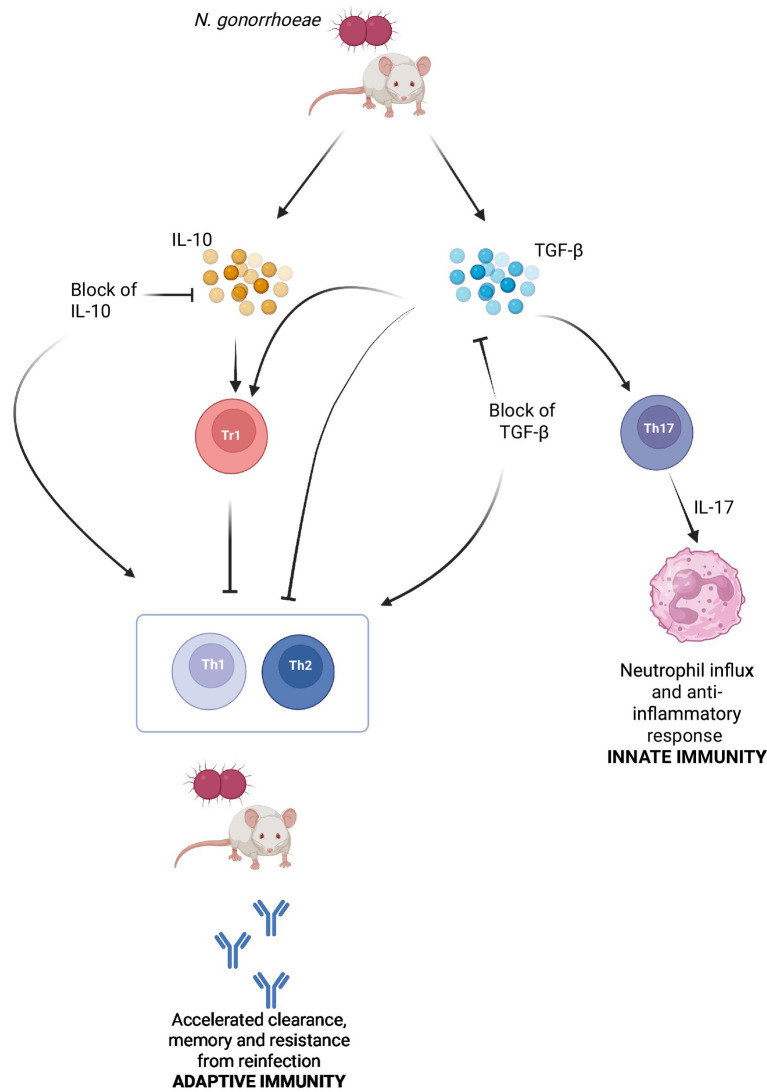
Gonococcal infection in the 17-β-oestradiol-treated mouse model. Gonococcal infection predominantly elicits a Th17-mediated immune response leading to an influx of neutrophils into the genital tract, which eventually facilitates gonococcal clearance. However, the Th17 response suppresses Th1 and Th2 immunity and relies on TGF-β signalling, which also enhances the development of T regulatory (Tr1) cells. Inhibition of TGF-β upregulates Th1 and Th2 immunity, accelerating bacterial clearance, promoting immunological memory, and conferring partial resistance to reinfection. Additionally, gonococcal infection induces IL-10 secretion, which drives differentiation of Tr1 cells and suppression of Th1 and Th2 responses. Blocking IL-10 enhances Th1 and Th2 immunity, improving bacterial clearance, immunological memory and the development of adaptive immunity.

Russell et al., at the University at Buffalo, used the mouse gonococcal infection model to demonstrate that *N. gonorrhoeae* triggers an immune response characterised by increased levels of IL-17A, associated with a Th17 cell and neutrophil influx at the site of infection, which eventually clears the infection but does not induce immunological memory (Figure 5) (Feinen et al., 2010). The Th17 response has been shown to rely, in part, on the induction of TGF-β, which suppresses Th1 and Th2 responses. Inhibition of TGF-β partially reverses this suppression, permitting activation of Th1 and Th2 responses, the formation of protective immune memory, and the production of gonococcal-specific antibodies in both blood and mucosal secretions (Liu and Russell, 2011). Moreover, TGF-β treatment of mice genetically deficient in either IL-12 (a Th1 cytokine) or IL-4 (a Th2 cytokine) demonstrated that accelerated clearance of primary gonococcal infection depends on Th1 responses but not Th2 responses (Figure 5). In contrast, resistance to secondary infection, indicative of immunological memory, necessitates both Th1 and Th2 responses (Liu and Russell, 2011). Th17 responses associated with gonococcal infection in the mouse model are linked to gonococcal LOS binding to TLR4 (Liu et al., 2012), while TGF-β is only partially induced by gonococcal Opa proteins (Liu et al., 2012).

Recent studies reveal that *N. gonorrhoeae* additionally induces the production of IL-10 and, consequently, the amplification of type 1 regulatory T (Tr1) cells (Figure 5) (Liu et al., 2014). IL-10 and Tr1 cells are potent suppressors of Th1 and Th2 immunity, and their induction further reinforces the Th17-dominated immune environment by actively constraining adaptive immune pathways required for durable protection (Taylor et al., 2006). Suppressing IL-10 and Tr1 cell activity enhanced Th1 and Th2 responses, leading to more rapid infection clearance and the development of immunological memory, as well as the generation of systemic and vaginal anti-gonococcal antibodies (Figure 5) (Liu et al., 2014).

In summary, *N. gonorrhoeae* triggers a Th17 response in mice that suppresses Th1 and Th2 immunity. Suppression of Th1 and Th2 immunity, which are linked to the development of immunological memory, depends on two cytokines, IL-10 and TGF-β. Blocking either TGF-β or IL-10 facilitates induction of immunological memory and improves protection in mice. How these findings apply to human infection is not known.

## 4 Treatment and prevention of gonococcal infection

### 4.1 The impact of antibiotic resistance on treatment strategies

Several oral and injectable antimicrobials have been used to successfully treat gonorrhoea (Unemo and Shafer, 2011). However, the increase of antimicrobial resistance to most available antimicrobials (including sulphonamides, penicillins, cephalosporins, tetracyclines, macrolides, and fluoroquinolones) among gonococci (Ohnishi et al., 2011; Yang and Yan, 2020) has resulted in the emergence of multi-drug resistant bacteria (Unemo and Shafer, 2011).

In the mid-1930, sulphonamides were the first antimicrobials used to treat gonorrhoea, but by 1944 many gonococcal clinical isolates had developed resistance to these (Unemo and Shafer, 2014). Penicillin then became the treatment of choice. However, resistance to penicillin emerged due to both chromosomal mutations and the spread of conjugative plasmids carrying β-lactamase genes, such as *pbla*, coding for the enzyme β-lactamase, which degrades penicillin. The combination of chromosomal mutations in genes like *penA* and the spread of β-lactamase-producing plasmids made penicillin resistance among gonococci widespread by the 1970s (Faruki et al., 1985; Unemo and Shafer, 2014).

In the 1960s, tetracycline was introduced to treat gonorrhoea. Tetracycline acts by binding to the bacterial ribosome and inhibiting protein synthesis. Resistance to tetracycline spread rapidly due to the acquisition of plasmids such as pConj, which carry genes encoding ribosomal protection proteins (Morse et al., 1986). These mechanisms confer resistance to tetracycline by either actively expelling the drug from the bacterial cell or by preventing tetracycline from binding to its target on the bacterial ribosome (Yee et al., 2023).

In response to tetracycline and penicillin resistance, antibiotics such as spectinomycin, an aminocyclitol which inhibits protein synthesis by targeting the ribosome, became a new treatment option. Within a few years, gonococci resistant to spectinomycin were isolated (Easmon et al., 1984). As a result, the antibiotic was largely discontinued as a recommended treatment for gonorrhoea. The fluoroquinolones, ciprofloxacin in particular, were widely employed to treat gonococcal infections from the mid-1980s until the 1990s, when increasing treatment failure was observed (Unemo and Shafer, 2011, 2014). Resistance to quinolones is mostly due to mutations in the *gyrA* and *parC* genes, encoding the bacterial enzymes DNA gyrase and topoisomerase IV, primary targets of these antimicrobials (Belland et al., 1994; Starnino et al., 2010; Unemo and Shafer, 2011). These mutations alter the structure of the enzymes, reducing the ability of the antibiotics to bind effectively, thus diminishing their capacity to inhibit DNA replication and cell division.

Azithromycin became a commonly adopted treatment for gonorrhoea in many countries after its discovery. Azithromycin is part of the macrolide class of antibiotics, which function by interacting with protein synthesis in the ribosome, leading to incomplete peptides (Douthwaite and Champney, 2001). However, resistance developed rapidly, driven by ribosomal mutations that affect the ability of the drug to bind. Additionally, overexpression of bacterial efflux pumps can actively expel azithromycin from the bacteria, further reducing its efficacy (Unemo and Shafer, 2014). Third-generation cephalosporins, such as cefixime and ceftriaxone, are current recommended monotherapy options for treating gonococcal infections. However, cefixime treatment failures have been recorded in numerous countries, including Japan, several European countries and Canada, and in South America (Yokoi et al., 2007; Ison et al., 2011; Ohnishi et al., 2011; Unemo et al., 2012; Allen et al., 2013).

Currently, in LMICs, symptomatic patients are often treated with doxycycline (Cehovin et al., 2018), despite extensive resistance in *Neisseria gonorrhoeae*. Tetracycline resistance is mediated by acquisition of the *tetM* plasmid, which encodes a protein that protects the ribosome from tetracycline binding, alongside overexpression of the MtrCDE efflux pump, reducing intracellular antibiotic concentrations, and chromosomal ribosomal mutations that impair antibiotic binding (Unemo and Shafer, 2011). In high-income countries (HICs), ceftriaxone remains the main option for gonococcal antimicrobial monotherapy. In response to concerns that gonorrhoea could become untreatable with monotherapy, Europe, the UK and USA previously recommended dual-antimicrobial therapy consisting of intramuscularly-administrated ceftriaxone and oral azithromycin or doxycycline (Ohnishi et al., 2011; Unemo and Shafer, 2011; Unemo et al., 2012; Lusti-Narasimhan et al., 2013; Lahra et al., 2014). However, the first documented failure of this dual regimen in pharyngeal gonorrhoea was reported in the UK in 2014 (Fifer et al., 2016), and since then, there has been an increase in isolates with resistance to multiple recommended antimicrobial classes, including decreased susceptibility to extended-spectrum cephalosporins. Resistance mechanisms in these isolates include alterations in *penA* as well as increased efflux pump activity and porin mutations that reduce antibiotic uptake (Unemo et al., 2012). Consequently, current guidelines from the WHO, UK and USA, now recommend higher-dose ceftriaxone monotherapy as first-line treatment (Unemo et al., 2019a; Workowski et al., 2021). More recently, novel antimicrobials have been developed to address the growing threat of extensively drug-resistant *N. gonorrhoeae*, including zoliflodacin and gepotidacin. Those oral agents inhibit bacterial DNA replication by targeting DNA gyrase and topoisomerase IV by binding to novel sites compared with fluoroquinolones. Both have demonstrated efficacy against multidrug-resistant strains, including those resistant to cephalosporins and macrolides (Taylor et al., 2018; Pascual et al., 2024; Ross et al., 2025).

The rapid development of antimicrobial resistance in *N. gonorrhoeae* and the limited pipeline of new antibiotics make effective treatment challenging. As a result, *N. gonorrhoeae* is a high priority pathogen for research and development of new treatment and prevention efforts and is included in the WHO priority list of AMR priority pathogens (World Health Organisation, 2024). From a global public health perspective, there is an urgent need for novel antimicrobials, alternative therapeutic agents, and preventive measures such as effective vaccines against *N. gonorrhoeae*. The WHO Global Health Sector Strategy on STIs has set the ambitious target of reducing gonococcal infection incidence by 90% by 2030 (Gottlieb et al., 2020; WHO, 2022). An effective gonococcal vaccine could play a significant role in reducing infection rates and AMR burden.

### 4.2 Historical gonococcal vaccine efforts

There are many limitations in the development of an *N. gonorrhoeae* vaccine. First, the absence of protective immunity following natural infection with gonorrhoea suggests that an effective vaccine needs to achieve a level of immunity that nature is not able to achieve. As a result, the lack of well-defined immune correlates of protection makes it challenging to evaluate the potential efficacy of new vaccines in the absence of field efficacy studies. Second, antigenic and phase variation of potential vaccine targets on the bacterial surface may render a single-antigen vaccine approach insufficiently protective. Last, the human-host specificity of the disease complicates vaccine development by limiting the availability of animal models for studying disease mechanisms and evaluating vaccine efficacy (Jerse and Deal, 2013; Jerse et al., 2014; Quillin and Seifert, 2018; Russell et al., 2019).

Since the 20th century, several attempts have been made to develop vaccines against gonorrhoea, although only two have ever been tested in humans as part of field efficacy trials (Maurakis and Cornelissen, 2022): a heat-killed, partially lysed whole cell-based vaccine (Greenberg et al., 1974; Greenberg, 1975) and a gonococcal purified pilin vaccine composed of a single pilus type (Boslego et al., 1991). Both failed to show efficacy. A third candidate based on a porin protein was abandoned after failing to induce protection in a CHIM study (Russell, 2018).

The heat-killed gonococcal vaccine comprised a mixture of heat-inactivated cells from two *N. gonorrhoeae* strains. This vaccine successfully elicited antibodies in most participants during a Phase 1 study (Greeberg et al., 1971). However, a subsequent Phase 3 trial, which used whole-cell preparations from three pooled *N. gonorrhoeae* strains, found no significant difference in infection rates between vaccinated and placebo groups (rate of confirmed gonococcal infection was 30% in the vaccinated group and 24% in the placebo group).

The second vaccine candidate, a purified pilus-based vaccine, successfully elicited both serum and genital anti-pilus antibody responses and showed efficacy in an initial CHIM study with a homologous *N. gonorrhoeae* strain (McChesney et al., 1982; Boslego et al., 1991). However, a subsequent placebo-controlled, double-blind trial conducted in Korea involving 3,250 volunteers found no significant difference in the cumulative incidence of confirmed *N. gonorrhoeae* infection between vaccinated and placebo groups, with an attack rate of 7.2% in the vaccinated group and 6.8% in the placebo group (Boslego et al., 1991). A subsequent CHIM study revealed that the vaccine did not provide protection against a heterologous strain with antigenically distinct pili (Tramont and Boslego, 1985).

In 1985, a third vaccine candidate, which consisted of outer membranes of a single strain of *N. gonorrhoeae* enriched with Por protein, was tested in a CHIM study. After intraurethral challenge with the same gonococcal strain as the vaccine strain, the study resulted in no significant difference in infection rates in the vaccinated group (49%) compared to the placebo (32%). However, the infection rates in both vaccinated and placebo groups were low. The proposed rationale behind the mechanism of action of the Por candidate vaccine was generation of an antibody response against Por, leading to complement-mediated bactericidal killing and/or opsonophagocytosis (Rice et al., 2017). However, the vaccine preparations contained additional outer membrane components, such as Rmp and LOS. As a consequence, vaccination elicited polyclonal antibody responses not only to Por, considered the protective vaccine antigen, but also to LOS and Rmp. At that time, it was not recognised that antibodies targeting these antigens, especially Rmp, could impact how other antibodies interact with the bacterium. Such potential interference possibly impaired complement-dependent killing activity of antibodies directed against Por and other bacterial antigens (Rice et al., 2017).

Although correlates of protection against gonococcal infections remain unknown, an effective gonococcal vaccine may need to trigger immune responses differing in quality and/or quantity from those generated by natural infection.

## 5 Gonococcal vaccines: the current situation

### 5.1 Meningococcal outer membrane vesicle vaccines are effective against gonorrhoea

The development of an effective vaccine against *N. gonorrhoeae* remains critical as the bacterium continues to acquire resistance to antimicrobial treatments. Despite past challenges with vaccine development, recently reviewed by Noori Goodarzi and Pourmand (2026), clinical evidence indicates partial effectiveness of *N. meningitidis* serogroup B (meningococcal B) outer membrane vesicle (OMV) vaccines against gonococcal infection (Petousis-Harris et al., 2017; Paynter et al., 2019; Abara et al., 2022; Wang et al., 2022; Bruxvoort et al., 2023) providing hope for the successful development of an effective *N. gonorrhoeae* vaccine.

A retrospective observational case–control study conducted in New Zealand following a mass vaccination campaign with MeNZB, a meningococcal group B OMV vaccine composed of detergent-extracted outer membrane vesicles (dOMVs) derived from the epidemic meningococcal strain NZ98/254, found cross-protection against *N. gonorrhoeae* with an estimated vaccine effectiveness of 31% (95% CI: 21–39%) (Petousis-Harris et al., 2017). This observation, together with subsequent studies showing decreased rates of gonorrhoea diagnosis following meningococcal B OMV vaccination in Cuba, Norway and Canada, indicates the potential of OMV vaccines to protect against gonorrhoea (Whelan et al., 2016; Longtin et al., 2017; Azze, 2019; Reyes Díaz et al., 2021).

The MeNZB vaccine is no longer available, but in 2013, the 4-component MenB vaccine 4CMenB (Bexsero, GSK) was licensed. 4CMenB includes dOMVs derived from the same New Zealand epidemic strain NZ98/254 and three immunogenic recombinant meningococcal outer membrane proteins, factor H-binding protein (fHbp), Neisserial adhesin A (NadA) and Neisserial Heparin Binding Antigen (NHBA) (Serruto et al., 2012). A 0.5 mL dose of 4CMenB contains 50 μg each of recombinant proteins and 25 μg of dOMV. The vaccine contains two additional meningococcal proteins, GNA2091 and GNA1030, which are fused to fHbp (GNA2091-fHbp) and NHBA (NHBA-GNA1030) respectively, in order to improve protein stability and enhance overall immunogenicity (Giuliani et al., 2006).

Several retrospective observational case–control studies have found that 4CMenB provides cross-protection against gonorrhoea in the United States, Italy and Australia. The immunological mechanism responsible for protection is not currently understood (Leduc et al., 2020; Abara et al., 2022; Wang et al., 2022; Bruxvoort et al., 2023). While meningococcal vaccines, such as 4CMenB, protect against meningococcal disease primarily through induction of bactericidal antibodies, this mechanism does not appear to be responsible for protection against *N. gonorrhoeae*. Sera from humans immunised with 4CMenB have been reported to lack complement-mediated killing activity against gonococcus (Beernink et al., 2019).

Despite *N. gonorrhoeae* and *N. meningitidis* being closely related and sharing between 80 and 90% sequence homology between their genomes (Tinsley and Nassif, 1996; Hadad et al., 2012), the major 4CMenB vaccine antigens, NadA, fHbp, and PorA, the latter being the principle MenB dOMV antigen, appear unlikely to have a significant role in providing cross-protection against gonococcal infections. The gene encoding NadA is absent in *N. gonorrhoeae* (Feavers and Maiden, 1998; Unemo et al., 2005), while the gene encoding fHbp is present and highly conserved in gonococcus, but the gonococcal fHbp protein is not surface-exposed and so unable to bind factor H (Hadad et al., 2012). The protection conferred by meningococcal dOMV vaccines against meningococcal infections is mainly attributed to the OMV protein PorA (Viviani et al., 2023). However, the gene homologous to PorA is a pseudogene in *N. gonorrhoeae*, and does not encode or produce a functional protein (Feavers and Maiden, 1998).

Conversely, the gene encoding NHBA in gonococcus shares over 68% homology with that of *N. meningitidis* and is highly conserved among gonococcal strains. Anti-NHBA antibodies induced by 4CMenB in humans are cross-reactive in both ELISA and Western blots and bactericidal against *N. gonorrhoeae* (Semchenko et al., 2019; Leduc et al., 2020; Tzeng et al., 2024). Therefore, NHBA may contribute to cross-protection. Finally, the two additional fusion protein components, GNA1030 and GNA2091, are present and well-conserved in most gonococcal isolates but not surface-exposed (Muzzi et al., 2013; Semchenko et al., 2019) and so are unlikely to contribute to protection.

Since MeNZB, which lacked recombinant proteins, was protective against gonorrhoea (Petousis-Harris et al., 2017), protection afforded by 4CMenB may be primarily attributable to the same dOMVs which are one component of 4CMenB. OMVs in 4CMenB and MeNZB include a core set of 22 proteins, which make up over 90% of the OMV content. Among these core OMV proteins, 20 out of 22 have sequence homologies in the gonococcal strain FA1090. Specifically, 16 of these homologues exhibit over 90% sequence identity (Semchenko et al., 2019). These high levels of sequence homology suggest that many of the core OMV proteins in the MeNZB vaccine may contribute to the cross-protection observed against gonococcal infections.

### 5.2 Recent progress towards a gonococcal vaccine

A range of technological approaches are currently being applied to the development of safe and efficacious vaccines against gonorrhoea. In recent years, several immunogenic proteins have emerged as promising vaccine targets and are currently under investigation in preclinical studies (Rice et al., 2017; Noori Goodarzi and Pourmand, 2026). These vaccine candidates include antigens that are immunogenic, surface-exposed, and conserved across a wide range of global strains. Proteins crucial for nutrient acquisition and metabolic processes, as well as those involved in antimicrobial resistance, are also being investigated, such as the iron acquisition systems TbpA/TbpB and the efflux pumps MtrCDE and FarAB-MtrE (Williams et al., 2024).

As a potential vaccine candidate, the gonococcal equivalent of NHBA, present in 4CMenB, is of interest due to its sequence conservation and wide expression across gonococcal strains. A recombinant NHBA candidate vaccine proved to be highly immunogenic in mice when combined with adjuvants such as Freund’s adjuvant or aluminium hydroxide, inducing serum bactericidal and opsonophagocytic killing (Semchenko et al., 2020).

Gonococcal surface transferrin receptor proteins (TbpA and TbpB) allow the bacterium to acquire iron from the host (described previously) and are critical for the survival of the bacterium in the human urogenital tract. For this reason, these proteins are considered promising vaccine antigens. TbpB is the surface-exposed lipoprotein component of the receptor, whereas TbpA is the integral membrane protein. Fegan et al. (2019) engineered TbpB as a scaffold to present conserved surface epitopes from TbpA. Mouse antisera generated against these hybrid antigens were effective in inhibiting the utilisation of transferrin by gonococci and accelerated clearance of infection following passive transfer in the mouse model of lower genital tract gonococcal infection.

MtrE, the outer membrane channel of the MtrCDE and FarAB-MtrE multidrug efflux pumps, has also been identified as a surface-exposed and conserved antigen. Immunisation of mice with purified MtrE adjuvanted with CpG induced bactericidal antibody responses and accelerated clearance of gonococcal infection in the murine model (Song et al., 2023). Additionally, gonococcal and meningococcal MtrE proteins exhibit ≥93% mean amino acid sequence similarity, suggesting that MtrE could serve as a cross-reactive antigen between *N. meningitidis* and *N. gonorrhoeae* (Marjuki et al., 2019).

Gonococcal LOS is an abundant, immunogenic and accessible target on the bacterial surface and, therefore, a potential vaccine candidate. However, the oligosaccharides of LOS can undergo phase variation, which raises concerns about their suitability as vaccine candidates. Despite this, the 2C7 LOS oligosaccharide epitope is widely expressed by gonococci (>95% of clinical isolates) during human infection. A peptide mimotope of the 2C7 LOS (MAP1) formulated with Th1-inducing adjuvants (Monophosphoryl Lipid A -MPLA, or glucopyranosyl lipid A-stable oil-in-water nanoemulsion - GLA-SE), was immunogenic, accelerating clearance of *N. gonorrhoeae* and lowering bacterial burden in the murine model of gonococcal infection. The same outcomes were observed with passive transfer of a monoclonal antibody against 2C7 (mAb2C7), suggesting that vaccine efficacy is dependent on the induction of functional antibodies (Gulati et al., 2013; Gulati et al., 2019b). Additionally, passive administration of mAb2C7 to C1q- and C9-deficient mice rendered the mAb2C7 ineffective, suggesting that complement activation is necessary for 2C7 vaccine function (Gulati et al., 2019a).

Current vaccine strategies have also focused on enhancing immunogenicity through the use of adjuvants and mucosal delivery routes. One approach involves microencapsulated IL-12 as an adjuvant to promote Th1 immune responses. The Th1 cytokine, IL-12, counteracts IL-10, a cytokine known to suppress Th1 responses by inducing Tr1 cells and polarising macrophages toward an M2 phenotype (Mills, 2012; Liu et al., 2014; Ortiz et al., 2015).

In the murine gonococcal infection model, intravaginal administration of microencapsulated IL-12 accelerated clearance of infection and resulted in resistance to re-infection with heterologous gonococcal strains, accompanied by the generation of anti-gonococcal serum and vaginal antibodies (Liu et al., 2013). However, administration to immunodeficient mice lacking either B cells or IFN*γ* during gonococcal infection failed to induce protection against re-infection, indicating the importance of both antibodies and IFNγ for effective immunity (Liu et al., 2018). Similarly, co-administration of IL-12 with OMVs from *N. gonorrhoeae* via the intranasal route accelerated gonococcal clearance and protected against heterologous gonococcal infection in the mouse gonococcal infection model. The candidate gonococcal OMV vaccine induced Th1-dependent immune responses, characterised by IFN-γ production and an increase in systemic, as well as mucosal, anti-gonococcal antibodies (Liu et al., 2023).

Bacterial ghosts, empty, membrane-bound shells of gram-negative bacteria created by the controlled lysis of bacterial cells, are also being explored as gonococcal vaccine candidates. Despite the absence of intracellular components, bacterial ghosts retain the antigenic profile of the original bacterium, making them attractive for antigen delivery (Tabrizi et al., 2004). Notably, *Salmonella*-derived ghosts have been employed to deliver PorB and Neisserial surface protein A (NspA), a highly conserved surface antigen. Immunisation of BALB/c mice with bacterial ghosts containing either PorB or NspA or a combination of the two elicited strong systematic antibody responses and cellular immune responses. The co-administration of ghosts containing PorB and NspA induced significantly higher bactericidal antibodies than single antigen administration (Jiao et al., 2018).

Through proteomics, the surface-exposed L-methionine-binding lipoprotein MetQ has been identified as a possible gonococcal vaccine candidate. MetQ is part of the ATP-binding cassette transporter system responsible for importing essential amino acids into the bacterium and is constitutively expressed in diverse gonococcal isolates (Zielke et al., 2014). When recombinant MetQ was formulated with CpG, it induced an immune response which enhanced bacterial clearance from the mouse genital tract and promoted production of antigen-specific antibodies in both serum and vaginal secretions (Sikora et al., 2020).

A proteomic analysis of the gonococcal cell envelope identified a number of novel candidate antigens, including BamA, a protein involved in membrane biogenesis, an LOS assembly protein, LptD, a protein transport system, TamA, and two other uncharacterised proteins, NGO2054 and NGO2139 (Zielke et al., 2016).

#### 5.2.1 Outer membrane vesicle vaccines

While the antigens described have shown promise in preclinical studies, it is important to note that a meningococcal OMV-based vaccine approach was found to be effective in retrospective studies at providing cross-protection against gonorrhoea. Therefore, pursuing the development of OMV-based gonococcal vaccines, which present a range of surface antigens, appears to be a particularly attractive approach. OMVs are being explored for their potential to protect against various bacterial pathogens, including *N. gonorrhoeae* (Micoli and MacLennan, 2020; Gu et al., 2026).

OMVs, such as those in 4CMenB, can be chemically extracted from whole bacteria using detergents, such as deoxycholate or sodium dodecyl sulphate, which increase OMV yield and reduce LPS content (detergent-extracted OMVs, dOMVs) (Micoli et al., 2020; Micoli and MacLennan, 2020). This approach potentially leads to compromised protein/lipoprotein integrity and contamination with cytoplasmatic material (Ferrari et al., 2006). More recently, bacterial strains have been genetically manipulated in order to reduce reactogenicity and/or increase production of OMVs, which have been termed native OMVs (nOMVs), mutant-derived OMVs (mdOMVs) and GMMA (Generalized Modules for Membrane Antigens) (Micoli and MacLennan, 2020). Inactivation of the *lpxL1* gene of *N. meningitidis* and *N. gonorrhoeae* reduces the number of lipid A acyl-chains from six to five, resulting in reduced stimulation of the TLR4/MD complex and a reduced inflammatory response (van der Ley et al., 2001; Fisseha et al., 2005; Sprong et al., 2011; Zhou et al., 2014; Micoli and MacLennan, 2020). nOMVs with attenuated LOS/LPS are usually formulated with aluminium hydroxide and have been shown to be safe in clinical trials (Marsay et al., 2015; Launay et al., 2017; Obiero et al., 2017).

Currently, all licensed OMV vaccines for humans have used detergent extraction as part of their production process and have been developed against *N. meningitidis* serogroup B (MenB). These include MeNZB, VA-MENGOC-BC™ and 4CMenB (Bexsero™). VA-MENGOC-BC™ was developed in Cuba and licensed in 1989. It was specifically designed to protect against a single meningococcal B strain type that was prevalent in that region at the time (Sierra et al., 1991). Other OMV vaccines have been developed to address specific *N. meningitidis* group B outbreaks in countries such as Norway and New Zealand (Bjune et al., 1991; Martin et al., 1998). These vaccines were also targeted at particular meningococcal group B epidemic strains and proved effective in addressing these outbreaks. These vaccines primarily induced an immune response against the immuno-dominant but highly polymorphic porin protein, PorA. As a result, the immune response induced is largely specific to the particular strain type used in the vaccine, potentially reducing efficacy against strains that differ in their PorA (Holst et al., 2005; Martin et al., 2006).

4CMenB (Bexsero) was developed to offer broader protection against various *Neisseria meningitidis* group B strains than MeNZB. Unlike MeNZB and VA-MENGOC-BC™, which consisted of OMVs, 4CMenB includes the same OMVs derived from the New Zealand epidemic MenB strain present in MeNZB and incorporates three recombinant proteins, as described previously. This broader antigenic profile aims to offer protection against a wide variety of MenB strains, overcoming limitations associated with strain-specific responses (Hadad et al., 2012). Despite meningococcal group B OMVs being largely strain-specific, they have been shown to provide cross-protection against gonococcal disease. Several prospective clinical trials are also ongoing, aiming to assess serological and cellular gonococcal-specific responses, as well as efficacy against gonorrhoea, following immunisation with 4CMenB (Belcher et al., 2023).

### 5.3 OMV vaccines against gonorrhoea

Due to the effectiveness of *N. meningitidis* serogroup B OMV-based vaccines against gonorrhoea, there is growing interest in developing new vaccines using OMV technology against *N. gonorrhoeae,* which have recently been reviewed by Gu et al. (2026).

Some efforts have focused on developing a universal vaccine that could provide broad protection against multiple *Neisseria* pathogens. At the same time, other initiatives have concentrated on creating gonococcal-specific OMVs. Some preclinical studies have explored meningococcal dOMVs derived from strains deficient in major outer membrane proteins, such as PorA, PorB, and Rmp (Matthias et al., 2022), as well as native meningococcal OMV with attenuated endotoxin and overexpressed fHbp (Beernink et al., 2019). This nOMV-fHbp candidate vaccine was immunogenic in mice, inducing anti-gonococcal serum bactericidal antibodies, a finding noteworthy since 4CMenB does not elicit bactericidal antibodies against *N. gonorrhoeae* in humans (Beernink et al., 2019).

Immunisation of mice with meningococcal dOMVs lacking major outer membrane proteins (OMP) enhanced gonococcal clearance when compared to wild type dOMVs and both induced serum and vaginal cross-reactive antibodies to gonococcus. IgG in serum from mice immunised with OMP-deficient dOMV bound to a wide range of gonococcal antigens by immunoblotting when compared with IgG from mice immunised with wild type dOMVs (Matthias et al., 2022). PorA is not expressed by the gonococcus, and gonococcal PorB is highly variable. Thus, antibodies against these antigens are unlikely to protective against different gonococcal strains.

Using a similar approach, Jones et al. (2024) engineered gonococcal OMVs by replacing the native PorB with its meningococcal counterpart. This modification increased the breadth of antigen-specific antibody responses to MtrE and MetQ by ELISA and to other antigens as detected by antigen microarrays, and resulted in a strong Th1 immune response, marked by elevated IgG2a and IFNγ production, traits considered crucial for gonococcal clearance.

The Gonococcal Vaccine Project (GVP) at the Jenner Institute is developing a gonococcal nOMV candidate vaccine, GonoVac. In early testing, mice immunised with a precursor of GonoVac cleared gonococci more rapidly than mice immunised with 4CMenB (Freixeiro et al., 2026), providing preclinical proof of concept.

GSK has tested a gonococcal nOMV candidate vaccine, NgG, based on the laboratory strain, *N. gonorrhoeae* FA1090 (Spinsanti et al., 2025), in a Phase 1/2 clinical trial, but discontinued development (Taylor, 2024) after failing to meet pre-defined endpoints (Trial ID: NCT05630859 source clinicaltrials.gov). The previously described IL-12 microencapsulated OMV vaccine, which has shown promising results in preclinical models, is also advancing through the development pipeline (Liu et al., 2023).

## 6 Summary and conclusions

*N. gonorrhoeae* is a human-restricted pathogen harbouring high levels of antimicrobial resistance with the capacity to evade immune recognition through antigenic and phase variation, complement inhibition, and manipulation of host immune pathways. Although infection induces local inflammation and detectable antibody production, it usually fails to confer protective immunity. Evidence from murine models suggests this is partly due to suppression of Th1 and Th2 responses, driven by immunomodulatory pathways involving IL-10 and TGF-β. In humans, adaptive immune responses tend to be limited unless infection spreads to the upper genito-urinary tract or causes severe disease.

Although historical vaccine attempts failed, recent findings offer optimism. The cross-protection observed with meningococcal OMV vaccines, along with the identification of conserved targets such as NHBA, MtrE, MetQ and the 2C7 LOS epitope, provides promising directions. Controlled human infection models and β-oestradiol-treated mouse models will be instrumental in unravelling immune responses and evaluating vaccine candidates, despite their limitations. Improvements in infection models and the application of immunomodulatory adjuvants may help overcome the suppressive immune environment characteristic of gonococcal infection.

Advancing a gonorrhoea vaccine necessitates overcoming pathogen immune evasion strategies and identifying antigens that induce functional, durable protective responses. This could be aided by the identification of correlates of protection which guide vaccine design toward sustained effective immunity.
